# A pilot study of at-home online cognitive screening prior to primary care wellness visits for older adults

**DOI:** 10.3389/fdgth.2026.1823218

**Published:** 2026-09-03

**Authors:** Louisa Thompson, Charles Eaton, Sarah Prieto, Molly Lawrence, Stephanie Czech, Jennifer Rosenbaum, David Anthony, Rabin Chandran, Lauren Kelly, Ivy Ngo, Arnold Goldberg, Caitlin Gillooly, Caroline Richardson, A. Rani Elwy, Richard N. Jones, Dorene M. Rentz

**Affiliations:** 1 Department of Psychiatry & Human Behavior, Alpert Medical School of Brown University, Providence, RI, United States; 2 Department of Family Medicine, Alpert Medical School of Brown University, Providence, RI, United States; 3 Department of Epidemiology, School of Public Health, Brown University, Providence, RI, United States; 4 Center for Healthcare Optimization & Implementation Research, VA Bedford Healthcare System, Bedford, MA, United States; 5 Department of Neurology, Massachusetts General Hospital, Harvard Medical School, Boston, MA, United States; 6 Center for Alzheimer Research & Treatment, Brigham and Women’s Hospital, Harvard Medical School, Boston, MA, United States

**Keywords:** aging, Alzheimer's disease, cognition, cognitive screening, early detection, primary care, remote assessment, dementia

## Abstract

**Background:**

Improved methods of cognitive testing are urgently needed for primary care settings faced with a growing older adult population and higher rates of dementia. We tested the feasibility and acceptability of a novel online cognitive test that can be completed at home prior to an annual exam.

**Methods:**

32 older adults completed testing at home on personal devices 1–4 weeks prior to an annual exam with their primary care provider. Additional cognitive screening was completed in-clinic to provide preliminary validation evidence. Completion rates were examined to assess feasibility, and patient acceptability was assessed via survey.

**Results:**

Completion rate was 78.5% for at-home testing online. Participants reported that they generally preferred at-home over in-clinic testing. At-home test performance was moderately correlated with the standard Montreal Cognitive Assessment completed in clinic.

**Conclusion:**

Findings provide initial support for the acceptability of self-administered online cognitive screening for older adults in primary care. Completion rates were slightly below a preset benchmark, suggesting that additional support may be needed to facilitate at-home testing in routine care. Further validation research is needed in larger samples including examination of digital measure convergence with clinical diagnoses and additional cognitive testing.

## Background

Improved detection and management of cognitive impairment is urgently needed in primary care, where approximately nine out of every ten older adults report receiving care (1). Recent estimates project that 42% of Americans over age 55 will eventually develop some form of dementia, with higher rates possible in women (2). Late onset Alzheimer's disease (AD), the most common cause of dementia, typically has a slow onset and progression, but the majority of cases still go undetected in primary care (3, 4). An estimated 90% of cases of mild cognitive impairment (MCI), often a precursor to AD or other dementias, go undiagnosed among Medicare beneficiaries, with higher rates missed among Black and Hispanic or Latino patients (4, 5).

Growing evidence in support of early interventions for modifiable dementia risk factors highlights the importance of identifying older adults at risk for cognitive decline as early as possible (6, 7). At the same time, attitudes toward dementia diagnosis are also changing. A recent survey found that 79% of Americans would prefer to receive a diagnosis while symptoms are minor and do not interfere with activities of daily living (12). Missed and/or delayed diagnoses not only heighten the risk of disease progression but also limit opportunities for patients to adopt beneficial lifestyle changes, access treatment, and participate in care planning (8, 9).

Commercially-available plasma biomarkers that detect the earliest stages of AD have recently been FDA approved to aid in the differential diagnosis of MCI and dementia; however, use guidelines indicate that these tests should only be used in the context of confirmed cognitive decline (10, 11). Unfortunately, existing paper-based cognitive screening tools are often insensitive for accurately detecting MCI, particularly in diverse older adults in primary care (12). This potentially creates a significant bottleneck in identifying appropriate candidates for biomarker testing. Several newer digital cognitive assessment tools have been shown to be feasible, sensitive to mild impairment, and highly reliable in older adults, but only a few have been validated for use in primary care (13–15). Moreover, given time constraints faced by primary care practitioners, there is a greater need to determine whether digital assessments could be self-administered remotely (i.e., at home) by older adults to initiate a diagnostic work up or supplement ongoing cognitive monitoring (16). With an estimated 88% of U.S. older adults using the internet regularly, now is the time to investigate digital alternatives to traditional testing (17, 18).

The Boston Cognitive Assessment (BOCA) is a 10 min online test of global cognition developed for clinical use in older adults. Strong correlations between BOCA and the Montreal Cognitive Assessment (MoCA) have been found in memory clinic patients and healthy controls (19, 20). Additionally, BOCA is sensitive to cerebral amyloid accumulation, a neuroimaging biomarker of AD (21), making it a promising candidate for earlier detection of cognitive impairment for primary care settings. We designed and conducted the present pilot study to evaluate the feasibility and acceptability of a remote cognitive screening intervention using the BOCA to detect cognitive impairment among older adult patients in primary care, with the following specific objectives: 1) Determine if remote testing with BOCA is feasible with minimal support based on test completion rates; 2) Survey the acceptability of digital tests among patients. Additionally, to collect preliminary validation evidence for the BOCA in this context of use, we examined associations between performance on the BOCA and in-clinic cognitive screening measures, including the paper-based MoCA (22), as a reference standard.

## Methods

### Recruitment & enrollment

Of approximately 80 primary care providers (PCPs) contacted via email invitation to partner in the study, we engaged five across three clinics in Rhode Island. Clinic A was a hospital-affiliated community clinic with two PCPs engaged in the study, clinic B was a Federally Qualified Health Center with one PCP engaged, and clinic C was a hospital-affiliated family medicine clinic with two PCPs engaged. Using electronic medical record (EMR) searches, we identified potential participants (age range of 55–85, no dementia diagnosis) from each partnering PCP's panel. We utilized a multipronged approach for recruitment to maximize insights for future research. Passive methods included mailings, MyChart messages (clinic A and clinic C only), and waiting room flyers. Direct recruitment included phone calls to MyChart users with preferences indicating willingness to be contacted for research, as well as calls to potential participants identified by the PCP.

Screening was conducted over the phone. Eligibility criteria required participants to be fluent in English. Exclusion criteria were existing dementia diagnosis or other neurological disease, a score of <13 on the telephone version of the MoCA (23), current substance use disorder, acute or unstable psychiatric illness, and motor or sensory conditions that would compromise test validity based on self-report during screening. Consent was collected electronically. Ethical approval for the study was obtained from the Butler Hospital Institutional Review Board (approval #1882523). Participant compensation of $20 was provided in the form of a gift card. Study data were stored on HIPAA-compliant Butler Hospital servers and accessed only by research staff.

### Cognitive assessment

#### Remote assessment

Remote assessment was conducted using the Boston Cognitive Assessment (BOCA), which participants completed using their own smartphone, personal computer, or tablet. The BOCA is a 10 min test of global cognition with subtasks assessing orientation, immediate and delayed recall, executive function, visuospatial ability, attention, and language scored for a total of 30 points (20). Testing within each subtask is preceded by a practice session. Alternate forms are given for repeat assessment. Additional test characteristics and psychometrics have been described previously (20). No personal identifying information is entered in the BOCA testing website, which uses a secure cloud server and encryption for data in transit.

Within one month of their annual PCP appointment, participants received a link to complete the BOCA via email. Up to two reminder emails or phone calls were made as needed. Participants were sent an email link to complete the BOCA a second time within one day of their appointment to examine test-retest reliability. Compliance for the first timepoint of BOCA testing was defined as completion of the task within one week and compliance for the second timepoint was defined as completion either day before or the day of the patient's PCP appointment.

#### In-clinic assessment

**Figure 1:**
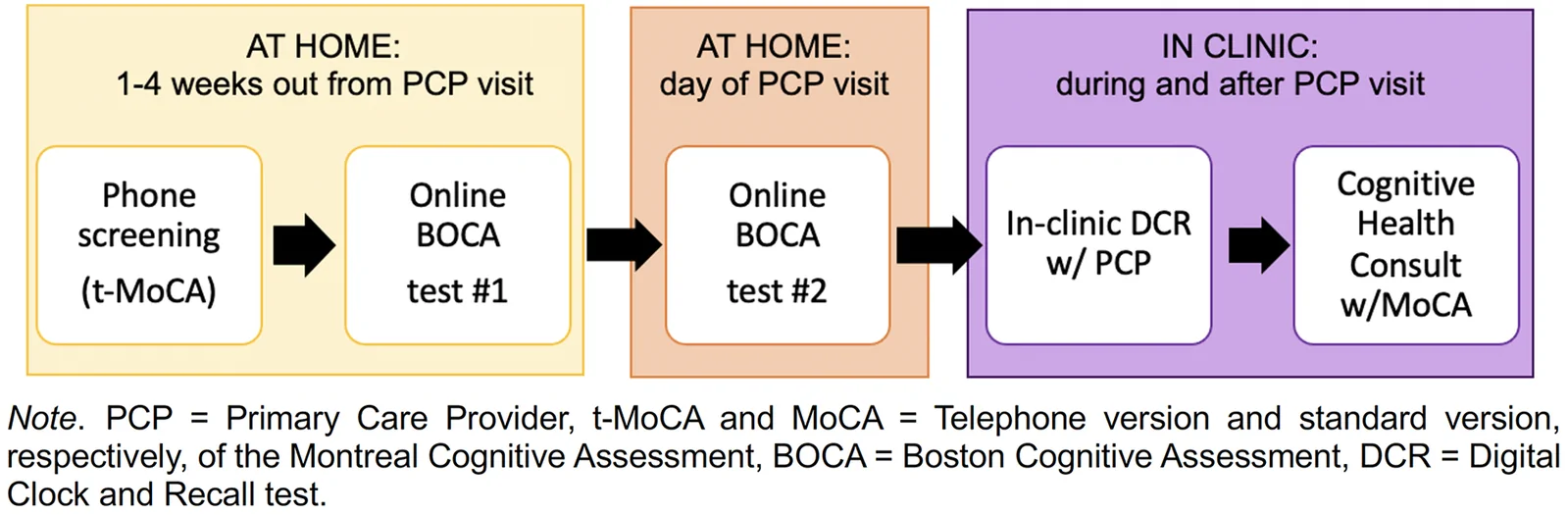
Protocol. PCP, Primary Care Provider; t-MoCA and MoCA, Telephone version and standard version, respectively, of the Montreal Cognitive Assessment; BOCA, Boston Cognitive Assessment; DCR, Digital Clock and Recall test.

At their PCP appointment, participants completed a comparison digital cognitive screening test, the Linus Health Digital Clock and Recall (DCR™), supervised by the PCP. The DCR is a 3–5 min assessment including 3-word learning, stylus-drawn clock, and 3-word recall. A cloud-based scoring algorithm calculates a total (0–5) and sub-scores (24–26). As the primary reference standard for validation, participants also completed the Montreal Cognitive Assessment (MoCA) administered by research staff at the end of their appointment as part of a brain health consultation (Figure 1). MoCA results were reviewed with participants and recommendations for maintaining brain health were provided as well as recommendations for follow-up, if needed. MoCA results were scanned to the participant's EMR with their permission. Impaired scores were communicated directly to the PCP and a follow-up referral to our memory clinic was offered.

### Survey & interview

Participants provided feedback via a 5–10 min online, REDCap survey. Likert scale survey items were developed to assess acceptability, including test preference (i.e., in-clinic versus at-home tests), perceived difficulty of each test, and usability (i.e., the BOCA website access and completion of testing with their personal device), as well as comfort level and stress associated remote versus in-clinic testing.

### Analysis

Wilcoxon rank-sum and chi-squared tests were used to examine potential differences in education, race, or employment status between enrolled participants and those screened but not enrolled. We set a benchmark completion rate of 80% to assess the feasibility of remote and in-clinic digital cognitive assessment completion, setting expectations slightly below rates reported in the literature for remote assessments deployed in non-clinical settings (27–29). Test-retest reliability was calculated for the BOCA administered at two timepoints (1–4 weeks apart). Convergent validity was tested using Spearman's correlations between the BOCA and the MoCA, and between the BoCA and DCR as a secondary convergent validity analysis. Area under the curve (AUC) analyses examined preliminary BOCA accuracy for distinguishing cognitive impairment on the MoCA using a cutoff of 26.

## Results

### Participants

A total of 48 older adults were screened for the study and 37 enrolled; however, five withdrew or were lost to follow-up before completing any study activities. Reasons for withdrawal, when given (*n* = 2), included not having enough time or needing to focus on other high priority health conditions. Overall attrition rate was 15.6% and the final enrolled sample size was *N* = 32. The sample was 56% female, 84% White and 16% Black. Mean participant age was 68 years (SD = 6.6) and mean years of education was 14.7 (SD = 2.4). Results indicated no significant differences in demographics between screened and enrolled participants.

### Protocol feasibility

#### Provider and patient engagement

Patient recruitment was limited to individuals in the care of our PCP partners. 613 mail invitations were sent to patients, yielding a 1.6% screening rate. 253 messages were sent via MyChart EMR system, yielding a 4.35% screening rate. Direct recruitment methods were more productive, with screening rates of 11.3% from 142 phone calls to MyChart users, and 30.3% from 33 direct PCP referrals. PCP direct referral was particularly useful at Clinic B, which did not use MyChart. There were no significant differences in recruitment method by participant demographics.

#### BOCA completion

Four participants who were enrolled less than five days prior to their PCP appointment were excluded from completion rate calculations. The completion rate for BOCA administered at time one (T1), 1–4 weeks in advance of the PCP appointment, was 78.5%. Six participants were non-compliant with T1 BOCA despite follow-up reminders. The completion rate was 66.7% for T2 BOCA testing within one day of the PCP appointment, with nine participants who were non-compliant. Reasons for non-compliance (when given) included not having time (*n* = 4), forgetting to complete the task (*n* = 3), and technical difficulties (*n* = 2).

### BOCA acceptability

Twenty-two participants completed the feedback survey. 50% preferred completing cognitive testing at home, compared to 10% who preferred in-clinic testing, and another 40% who said they preferred to do both at-home and in-clinic testing. 78% reported feeling more comfortable completing cognitive testing at home, while the other 22% said they felt more comfortable in the clinic. The in-clinic testing was rated as more difficult (65%) compared to the at-home test. All reported that they would be willing to complete the at-home test again in the future. At-home testing was most often completed in the morning using a smartphone (48%) versus a computer (28%) or tablet (24%).

### Preliminary BOCA validity

Preliminary BOCA test-retest reliability between the T1 and T2 timepoints was good (*r* = .81, 95% CI [.54–.93], *n* = 17, mean time difference = 15.5 days, range 4–33 days). Subsequent analyses examined only the first BOCA instance for each participant collapsed across T1 and T2. BOCA total scores were moderately correlated with the MoCA (*r* = .59, 95% CI [.28–.79], *p* < .001) and strongly correlated with the DCR (*r* = .71, 95% CI [.45–.86], *p* < .001). Mean BOCA score was 27.4, SD = 3.2, while mean MoCA score was 26.5, SD = 2.6. An exploratory area under the curve (AUC) analysis indicated a BOCA total score AUC = .80, 95% CI [0.60–1.0] to distinguish cognitive status (Figure 1), though estimates were imprecise, as indicated by the wide confidence interval, suggesting that the result should be interpreted cautiously. A cutoff score for identifying impairment has yet to be established for BOCA. Based on this preliminary ROC curve analysis, a cutoff of 24 would maximize sensitivity at .91, with a specificity of .71. This cutoff would result in a high negative predictive power but poor positive predictive power in clinical settings with similar prevalence rates of cognitive impairment (∼15%), suggesting that the utility of the BOCA would primarily be in identifying individuals to prioritize for in-clinic confirmatory screening.

## Discussion

Our results indicate that unsupervised online testing with BOCA at home is an acceptable approach to cognitive screening for older adults in primary care. However, the BOCA completion rate of 78.5% did not quite meet our 80% benchmark despite the provision of reminders, suggesting that the use of BOCA or other similar tools in routine care would likely require significant support resources to facilitate completion. Future deployment of at-home, unsupervised digital tests may benefit from a designated coordinator who could enlist a family member or assist with technology support, as needed. Remote digital assessments would also benefit from integration with electronic medical records (EMR) or artificial intelligence (AI) tools for setup, reminders, and monitoring. Indeed, recent literature has also identified workflow compatibility as an important feature for digital tool selection among providers (30). While EMR integration is advancing and has the potential to enhance implementation, scalable protocols that do not rely on EMR are still needed for many resource-limited settings (31).

Completing the digital cognitive testing at home was widely viewed as acceptable to patients and there were few technical challenges, suggesting that online tests completed via simple URL links are practical for remote use across a variety of device types. Although most patients said they preferred to do testing at home and were more comfortable with testing at-home, 40% said that they would prefer to complete both at-home and in-clinic testing, suggesting patients may also see value in repeating a test in-clinic with their provider.

The BOCA demonstrated preliminary validity, showing a moderate correlation with the MoCA, which is consistent with the prior BOCA literature limited to secondary care settings (21, 26). Further validation in larger samples is needed to examine convergent validity with in-depth cognitive testing and clinical workup. BOCA showed preliminary evidence for discriminability of MoCA-defined cognitive status, though estimates were imprecise. The sensitivity and specificity of our preliminary cutoff score indicates that BOCA testing prior to an annual appointment could be beneficial for first line screening, with positive results followed by confirmatory testing in-clinic. This approach could allow primary care providers to identify at-risk individuals prior to annual visits, enabling targeted follow-up while reducing in-clinic testing demands. Scalable, home-based testing could be particularly valuable given time constraints in primary care and the increasing need for early detection for the timely initiation of disease-modifying treatments for Alzheimer's disease. Providing participants with their screening results and educational materials to support brain health awareness was a strength of this study which can be extended to larger implementation efforts.

Examining acceptability and barriers to cognitive testing, including digital and remote approaches, should be explored in future implementation studies. Interestingly, given the minimal facilitation provided for this pilot study, providers initially chose to administer the DCR themselves despite the option to delegate to clinic support staff, citing high staff burden and turnover. These insights underscore the potential advantage of at-home testing and need to design tools and support workflows that minimize added workload for the full care team. For patient engagement, direct referrals from PCPs proved the most effective recruitment method, while passive approaches were less successful. These findings reinforce the importance of trusted provider-patient relationships to facilitate patient education and engagement in cognitive testing.

The small and geographically limited sample likely constrains the generalizability of our findings. Individuals who we engaged in the study were likely more digitally literate and motivated than typical primary care populations. Participants were more highly educated and less racially diverse than state averages, which may have influenced performance and validity outcomes. The MoCA itself is a screening tool with several limitations and is therefore not sufficient for diagnosing cognitive disorders (18). While the MoCA cutoff of <26 was appropriate for our sample, alternative cutoffs or reference standards may be necessary for more racially and ethnically diverse populations and individuals with lower levels of education, who are critical to engage in digital health research (32, 33). While the MoCA offers an initial benchmark for convergent validity, more in-depth multimodal validation studies comparing novel digital tools with more extensive cognitive testing, disease biomarkers, and functional assessments are needed. Finally, given the frequency of comorbid and complex medical conditions among older adults in primary care, further examination of BOCA accuracy accounting for comorbidities, particularly those known to affect cognition, will be a critical for more in-depth validation studies.

Given our small sample, we did not examine variability in performance or completion rates on the basis of digital device type or operating system. The BOCA has previously been developed and validated for use across multiple platforms and screen formats and does not include assessments of reaction time or processing speed which may be more sensitive to operating system differences. Consideration must also be given to the limitations inherent to remote and unsupervised testing, which is potentially susceptible to noise due to environmental variability, distractions, or interference. For these reasons, we recommend that remote assessments be repeated or confirmed with other assessments in the clinic for individuals with low scores or reported cognitive concerns.

Although evaluating clinical outcomes was not a focus of this study, the involvement of patients in the study successfully facilitated several referrals for further evaluation to our memory clinic or with a neuropsychologist. Several participants with low or borderline scores were referred for further evaluation, and some were identified as having potentially treatable contributing conditions. These cases highlight the potential clinical value of integrating cognitive screening into routine care. A focus of future research will include assessing whether early detection via digital tools improves rates of diagnosis and dementia care outcomes.

In sum, our findings provide preliminary support for the feasibility and acceptability of remote digital cognitive assessments in primary care but highlight the necessity of supportive infrastructure to ensure successful implementation. Continued research with larger, more diverse populations and in varied practice settings will be essential to optimize these tools for widespread adoption.

## Data Availability

The raw data supporting the conclusions of this article will be made available by the authors, without undue reservation.
